# Factors influencing fruit cracking: an environmental and agronomic perspective

**DOI:** 10.3389/fpls.2024.1343452

**Published:** 2024-02-16

**Authors:** Paolo La Spada, Eva Dominguez, Alberto Continella, Antonio Heredia, Alessandra Gentile

**Affiliations:** ^1^ Dipartimento Agricoltura, Alimentazione e Ambiente (Di3A) - Università degli Studi di Catania, Catania, Italy; ^2^ Departamento de Mejora Genética y Biotecnología, Instituto de Hortofruticultura Subtropical y Mediterránea La Mayora, Universidad de Málaga - Consejo Superior de Investigaciones Científicas, Málaga, Spain; ^3^ Departamento de Biología Molecular y Bioquímica, Instituto de Hortofruticultura Subtropical y Mediterránea La Mayora, Universidad de Málaga - Consejo Superior de Investigaciones Científicas, Málaga, Spain

**Keywords:** cracking, cuticle, environmental factors, agronomic factors, precision farming

## Abstract

Fruit cracking, a widespread physiological disorder affecting various fruit crops and vegetables, has profound implications for fruit quality and marketability. This mini review delves into the multifaceted factors contributing to fruit cracking and emphasizes the pivotal roles of environmental and agronomic factors in its occurrence. Environmental variables such as temperature, relative humidity, and light exposure are explored as determinants factors influencing fruit cracking susceptibility. Furthermore, the significance of mineral nutrition and plant growth regulators in mitigating fruit cracking risk is elucidated, being calcium deficiency identified as a prominent variable in various fruit species. In recent years, precision farming and monitoring systems have emerged as valuable tools for managing environmental factors and optimizing fruit production. By meticulously tracking parameters such as temperature, humidity, soil moisture, and fruit skin temperature, growers can make informed decisions to prevent or alleviate fruit cracking. In conclusion, effective prevention of fruit cracking necessitates a comprehensive approach that encompasses both environmental and agronomic factors.

## 1 Introduction

Fruit cracking is a disorder prevalent among various fruit crops and vegetables. Cracking has a detrimental impact on the quality of fleshy fruits from various species (Brüggenwirth and Knoche, 2017; Lara et al., 2019; Schumann et al., 2019) not only due to poor fruit appearance but also to shelf-life reduction, and increased susceptibility to infections by fungi and other pathogens, hence causing significant losses in the fresh market (Khadivi-Khub, 2015; Butani et al., 2019; Wang et al., 2021). Numerous factors and mechanism have been shown to contribute to fruit cracking susceptibility such as agronomic factors (e. g., nutrition imbalance, unmanaged irrigation), environmental factors (e.g., high temperature, high relative humidity), genetic factors as well as the physiology and biochemistry of the species (Correia et al., 2018; Kaur et al., 2019). These factors do not only contribute to fruit cracking, but they also have the potential to modify the composition and morphology of the cuticle. The plant cuticle is a supramolecular composite that can be considered as a modification of the epidermal cell wall (Domínguez et al., 2011). It is mainly constituted by the polyester cutin, waxes and discrete amounts of plant phenolics. The plant cuticle is also subjected to changes due to developmental, environmental and hormonal factors (Tafolla-Arellano et al., 2018). The cuticle is a primary barrier against water and solute transport and fruit rot pathogens, responding to environmental conditions like water deficit, changes in relative humidity, temperature or light intensity (Knoche, 2019; Lara et al., 2019). It is strongly related to fruit cracking resistance due to the provides mechanical support provided (Zarrouk et al., 2018).

Two hypotheses have been developed to try to explain the mechanism of fruit cracking: the critical turgor and the zipper model. In both of these models there is a strong influence of environmental and agronomic factors. Critical turgor hypothesis initially investigated in grapes (*Vitis vinifera* L.) and reported by (Considine and Kriedemann, 1972) suggests that the causes of fruit cracking are moisture accumulation due to low evaporative demand. This can be worsened by none or poor pruning technique, thus resulting in an increasing in fruit internal pressure that can lead to fruit cracking. More recently Knoche (2019) proposed the zipper model hypothesis, which was developed in sweet cherries (*Prunus avium* L.). This model outlines how tension stresses in the fruit cuticle during fruit ripening lead to the development of microcracks, which can then be aggravated by high relative humidity and water deposition on the surface. These cuticle microcracks formed during the ripening stage, allows water uptake of the outer mesocarp cells with higher negative osmotic potential, subsequent bursting, and leak of the cell malic acid causing the plasmolysis of skin cell that leads to cell wall swelling. This continuous cycle of events decreases the fracture pressure and causes visible cracking. Chang and Keller (2021) proposed that, in grape cracking both the critical turgor hypothesis and the recent zipper model hypothesis should be considered complementary.

In this mini review we will discuss how fruit cracking is strongly influenced by agronomic (mineral nutrition, plant growth regulators, bagging technique, irrigation and post-harvest management) and environmental factors (relative humidity and temperature). We will also highlight and emphasize the potential of machine learning and precision agriculture and how these techniques could provide some predictive models for the effective handling of the environmental factors, as well the vulnerabilities in agronomic management or soil deficiencies.

## 2 Environmental factors

Environmental conditions have a fundamental role in shaping the growth and quality of fruit production, and the effects are observed during developmental stages (Jiang et al., 2010). Given the current situation of global climate change, and the postulated role of the cuticle in cracking, it’s essential to grasp the biophysical characteristics of the cuticle and comprehend how environmental factors such as temperature, UV radiation, and relative humidity influence both the formation of the cuticle and its functional characteristics and properties (Domínguez et al., 2011; Singh et al., 2020).

Matas et al. (2005) demonstrated that temperature and relative humidity (HR) influence the mechanical characteristics of the isolated cuticle suggesting that environmental factors can have a crucial role in cracking. Chen et al. (2020) have reported that in blueberries (*Vaccinium* spp.) there is a positive relationship between fruit water loss and the amount of wax esters; this relationship, however, wasn’t found in grape (Fernández-Muñoz et al., 2022).

Fruit cracking intensity has been reported to vary considerably among years, pointing to a significant relationship with environmental factors (Aliviela et al., 1994). Several authors have reported the influence of environmental conditions in causing and enhancing fruit cracking, e.g. Choi et al. (2015) reported that fruit cracking in pear (*Pyrus communis* L.) cv Mansoo was influenced by the photoperiod during the fruit development; with a shorter sunlight exposure fruit cracking was higher than with a more prolonged sunlight exposure, this is likely since both gene expression and plant metabolism are influenced by the amount and quality and the angle of interception of light that plants receive directly impacts fruit growth, overall productivity, and susceptibility to disorders (Lin et al., 2017). Regarding temperature, Seo et al. (2022) reported in pear that unexpected drops in temperature during the blossoming phase can result in a decrease of on fruit production and trigger the occurrence of fruit cracking. Also, differences between day and night temperatures were shown to stimulate fruit cracking in pomegranate (*Punica granatum* L.) (El-Rhman, 2010). Simon (2006) reported that in sweet cherries (*P. avium* L.), there was a linear increase in fruit cracking with temperature. More recently, Singh et al. (2020) clearly showed that the water stress caused by high environmental temperature measured as leaf relative water content and water potential, directly impacted the extent of cracking in pomegranate fruits. On the other hand, Choi et al. (2020) noted that fruit cracking severity in pear cv Whasan increased in orchards that underwent rapid change from dry soil condition to heavy rainfall. The rapid fluctuation of soil moisture increased the water potential in the fleshy cells and led to higher turgor pressure in the cork and stone cells, thus inducing fruit cracking. Moreover, heavy rainfall during the period of rapid fruit growth was correlated with cracking in grape (Clarke et al., 2010), and in apricot (Gülsen et al., 1995). In mandarin (*Citrus reticulata* Blanco) cv Nova, grown during dry hot summers in the Mediterranean basin, seasonal water deficit followed by heavy rain during the cell enlargement stage was also linked to fruit cracking (Almela and Agustí, 1990).

## 3 Agronomic factors

All commercial orchards require a correct balance of both macro and micronutrients to achieve satisfactory growth, development, and productivity (Seo et al., 2022).It has been reported that the mineral nutritional status of crop and, therefore, of fruits is closely related to fruit cracking (Shi et al., 2022). Among all the mineral elements necessary for the growth of orchards, calcium participates in numerous vital processes due to its role in the structural integrity and stability of the cell walls and middle lamella. Calcium is a connecting link between pectin molecules, enhancing the robustness of cell membranes by strengthening phospholipid bonds. Furthermore, it acts as a secondary messenger in stress signal transduction pathways (Ranty et al., 2016; Yu et al., 2020).

Calcium is not the only mineral element to play a key role in physiological processes, potassium, zinc, boron, copper, manganese, and molybdenum also govern fruit growth and development. A deficiency in any of these nutrients during the growth phase can lead to fruit cracking (Sheikh and Manjula, 2012). Moreover, it was reported that copper hydroxide at low concentrations or in combination with calcium hydroxide, significantly reduced fruit cracking (Brown et al., 1995). On the other hand, calcium deficiency has been identified to be one of the main causes of fruit cracking in grape (Yu et al., 2020), lemons (*Citrus limon* L. (Osbeck)) (Devi et al., 2018), litchis (*Litchi chinensis* Sonn.) (Martínez Bolaños et al., 2017), pomegranates (Davarpanah et al., 2018), and many other fruit crops (Khadivi-Khub, 2015; Fischer et al., 2021).

Plant growth regulators (PGRs) have been used for years for different purposes in orchards, from fruit thinning (Davis et al., 2004) to control fruit shape and size in several crops (Ginzberg and Stern, 2016; Continella et al., 2023). It has been reported that high endogenous levels of gibberellic acid (GA) are associated with a thicker cuticle and lower incidence of russeting in apple (Eccher, 1986; Eccher and Hajnajari, 2006). External application of PGRs in orchards has also been shown to reduce russeting or cracking incidence by affecting the epidermal tissue (Ginzberg and Stern, 2016). Stern et al. (2013) and Ginzberg et al. (2014) suggested that application of a mixture of PGRs (i.e., mixtures of 6-benzyladenine (BA) and GA4 + 7) may affect skin characteristics in apple cv Pink lady and reduce calyx-end cracking. Regarding this topic, some apparently contrasting results have been reported. In apple fruits (*Malus domestica* (Suckow) Borkh), it was suggested that GA_4 + 7_ treatment did not affect the amount or rate of cutin or wax deposition, but rather the epidermal and hypodermal tissues (Knoche et al., 2011). Conversely, in tomato fruit, GA increased cuticle mass per fruit surface and reduced micro-cracking (Knoche and Peschel, 2007). These apparently conflicting results may have been due to the use of different formulas or concentrations of the GA applied as well as different timing of the treatment with respect to the fruit phenological stage. Thus, it is important to indicate that the role of PGRs is linked to the stage of development and formula concentration, and these parameters should be determined for each individual species (Ginzberg and Stern, 2016).

Among phytohormones, ethylene plays an important role in fruit ripening, but also plays a major role in various developmental processes such as seed germination, flowering, organ senescence, programmed cell death and the response to biotic and abiotic stresses (Lin et al., 2009; Liu et al., 2015; Gao et al., 2020). Both in pre-harvest and post-harvest, ethylene seems to have an indirect role in affecting fruit cracking: indeed, in Charentais-type melon cv ‘Vedrantais’ producing high ethylene rates, stem-end splitting was already noticed 2h after the ethylene peak (Fernández-Trujillo et al., 2013). In post-harvest, among the methods that can be used to reduce the incidence of cracking, 1-methylcyclopropene (1-MCP), which inhibits ethylene perception by binding receptors to form an ethylene-receptor complex, delayed fruit ripening, significantly reducing the susceptibility to cracking in apples cv. ‘Royal Gala’ (Lee et al., 2016).

The role of ethylene is highlighted also by (Santos et al., 2023) that reported its connection with various cracking-related genes included in the ethylene production pathway such as*: aA, SS, TLP, ACCS, H1* in litchi (Wang et al., 2019b, Wang et al. 2019a), *ACO, ACS* in litchi and sweet cherry (Wang et al., 2019a; Michailidis et al., 2021) and *ER* in tomatoes (Xue et al., 2020). These genes associated with ethylene biosynthesis could contribute to the development of hybrids that are not only resistant to cracking, but also more suitable for a longer shelf-life: (Liao et al., 2020) targeted in watermelon the ethylene-sensitive transcription factor 4 (ClERF4) as the causative gene relevant to rind hardness, an important factor for genetic improvement in cracking resistance. As a whole, numerous scientific evidences underlined the reduction of ethylene production as an efficient tool during post-harvest management.

Irrigation is another agronomic factor crucial for fruit production and preventing cracking. It was demonstrated that water stress followed by a high volume irrigation could cause rapid meristematic growth and increase fruit cracking in pomegranate (Galindo et al., 2014) and apple (Goodwin et al., 2022). A good irrigation management, including sustained deficit irrigation (SDI) and deficit irrigation (DI) can be useful to reduce fruit cracking without losing yield, as reported by (Blanco et al., 2022) in sweet cherries.

To minimize fruit quality deterioration within the orchards, farmers employ a set of agricultural practices as means of safeguarding their fruits from various factors that could cause damages. One of these practices is the “bagging” technique to protect fruits from direct sunlight and direct heat that can cause excessive evapotranspiration from the fruit surface, resulting in excessive moisture loss. This technique is widely used to improve fruit quality with a broad range of different types of materials as shown by Ali et al. (2021). Singh et al. (2020) reported several scientific articles in pomegranate related to the reduction of fruit cracking using the bagging technique. Additionally in pear and apple, fruit bagging at the early stage of growth was shown to prevent fruit cracking (Choi et al., 2015) and Kasai et al. (2008) noted that bagging significantly reduced fruit cracking in apple cv Fuji.

## 4 Precision farming and machine learning

Modern agriculture benefits significantly from precision farming and monitoring systems. These systems play a pivotal role in optimizing various aspects such as water use efficiency (WUE), weed control, fertilizer utilization, and early identification of conditions favoring fungal infections (Monteiro et al., 2021). The integration of machine learning tools has become increasingly prevalent across different fields. For example, machine learning algorithms, including K-Nearest Neighbor (KNN), Random Forest (RF), Support Vector Machine (SVM), and Artificial Neural Network (ANN) are currently being employed to classify fruit fly species based on morphometric data (Salifu et al., 2022). Clearly, the use of these techniques are having an impact on fruit quality that is expected to increase in future years.

Nowadays, monitoring the parameters useful to enhance fruit production as well as to obtain high-quality fruits is possible. This kind of monitoring can be divided into two groups:

1. Epigean parameters: air temperature, relative humidity, radiation, sum of daily radiation, wind speed, wind direction, rain counter, evapotranspiration calculation, and dew point.
2. Hypogean parameters: volumetric water content of the soil, tensiometers (tension of soil water), multi-level soil humidity, and temperature probe.

Recently, RGB-thermal cameras were applied to monitor fruit skin temperature in sweet cherries (Osroosh and Peters, 2019). On the other hand, (Ranjan et al., 2022) implemented two cultivar-specific wetness prediction models on sweet cherries cv Skeena and Selah. In this work, the authors used a combination of microclimate sensing for weather data, and RGB-thermal camera to obtain thermal images and precise data on the fruit wetness. From a machine learning point of view, there are multiple studies that deal with analyzing biotic and abiotic stresses in crops, using the ICQP (identification, classification, quantification, prediction) paradigm well descripted by (Rico-Chávez et al., 2022). These works on precision farming and machine learning open the possibility of developing a tailored-made model for each species and integrating the resulting models in decision support systems for agriculture.

## 5 Conclusions

In conclusion, fruit cracking is a complex physiological disorder that affects the quality and marketability of various fruit crops and vegetables, is influenced by a multitude of factors. We focused on environmental and agronomic factors highlighting the significant impact of environmental factors such as temperature, relative humidity, and light exposure on fruit cracking. Agronomic factors, particularly mineral nutrition, and plant growth regulators (PGRs) and post-harvest management, also play a crucial role in fruit cracking susceptibility. Calcium deficiency has been identified as a leading cause of fruit cracking in various fruit species. Proper nutrient management and the judicious use of plant growth regulators can mitigate the risk of cracking in orchards.

In the last five years, machine learning, precision farming and monitoring systems have emerged as valuable tools for managing environmental factors and optimizing fruit production. By closely monitoring parameters such as temperature, humidity, soil moisture, and fruit skin temperature, growers can make better decisions to prevent or reduce fruit cracking. Tailored models and decision support systems offer promising avenues for improving fruit quality while minimizing losses due to disorders like fruit cracking.

**Figure 1:**
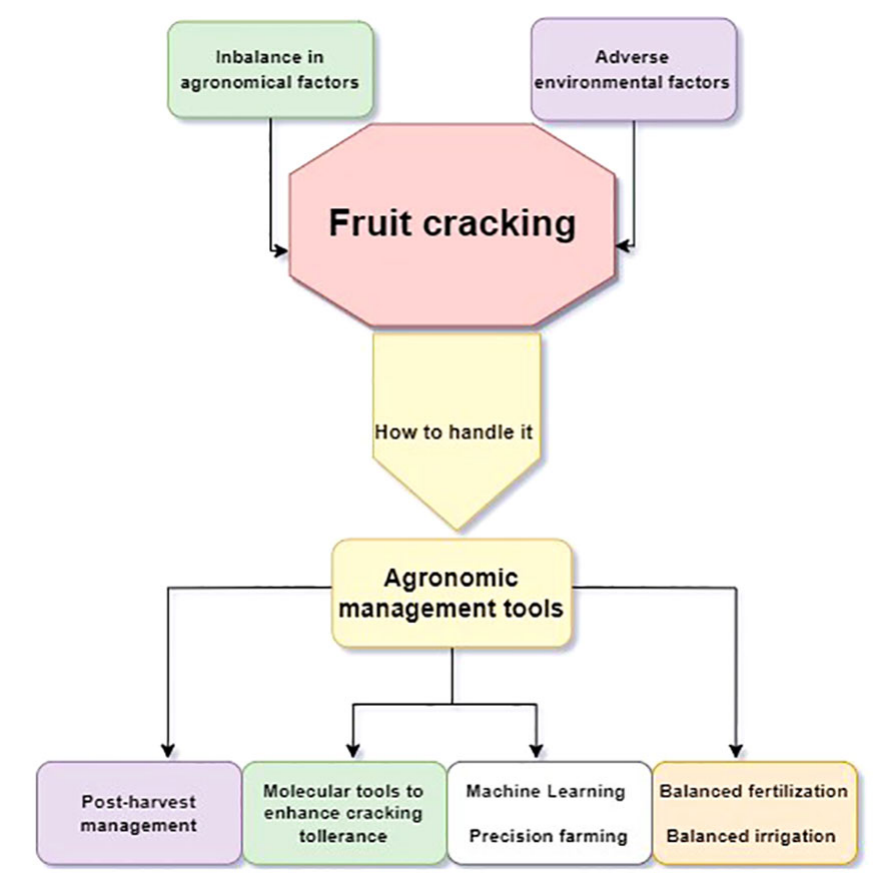
Schematic diagram of the agronomic approach to manage fruit cracking.

As reported in **Figure 1** fruit cracking prevention requires a holistic approach that considers both environmental and agronomic factors shaped by the choice of genotype and the scion-rootstock interaction; with the growing challenges induced by climate change, adopting precision farming practices and harnessing technology to monitor and predict the predisposing conditions for fruit cracking will be essential for ensuring high-quality fruit production, to develop effective strategies to minimize economic losses.

## Author contributions

PS: Conceptualization, Writing – original draft, Writing – review & editing. ED: Conceptualization, Writing – review & editing, Funding acquisition. AC: Funding acquisition, Writing – review & editing, Supervision. AH: Conceptualization, Funding acquisition, Writing – review & editing. AG: Funding acquisition, Supervision, Writing – review & editing.
